# LZTR1 deficiency exerts high metastatic potential by enhancing sensitivity to EMT induction and controlling KLHL12-mediated collagen secretion

**DOI:** 10.1038/s41419-023-06072-9

**Published:** 2023-08-25

**Authors:** Taiki Abe, Shin-ichiro Kanno, Tetsuya Niihori, Miho Terao, Shuji Takada, Yoko Aoki

**Affiliations:** 1 Department of Medical Genetics, Tohoku University School of Medicine, Sendai, Japan; 2 Division of Dynamic Proteome, Institute of Development, Aging, and Cancer, Tohoku University, Sendai, Japan; 3 Department of Systems BioMedicine, National Research Institute for Child Health and Development, Tokyo, Japan

**Keywords:** Metastasis, Ubiquitylation, Ubiquitylation, Protein-protein interaction networks, Kinases

## Abstract

Leucine zipper-like transcriptional regulator 1 (LZTR1), a substrate adaptor of Cullin 3 (CUL3)-based E3 ubiquitin ligase, regulates proteostasis of the RAS subfamily. Mutations in *LZTR1* have been identified in patients with several types of cancer. However, the role of LZTR1 in tumor metastasis and the target molecules of LZTR1, excluding the RAS subfamily, are not clearly understood. Here, we show that LZTR1 deficiency increases tumor growth and metastasis. In lung adenocarcinoma cells, LZTR1 deficiency induced the accumulation of the RAS subfamily and enhanced cell proliferation, invasion, and xenograft tumor growth. Multi-omics analysis to clarify the pathways related to tumor progression showed that MAPK signaling, epithelial-mesenchymal transition (EMT), and extracellular matrix (ECM) remodeling-related gene ontology terms were enriched in LZTR1 knockout cells. Indeed, LZTR1 deficiency induced high expression of EMT markers under TGF-β1 treatment. Our search for novel substrates that interact with LZTR1 resulted in the discovery of a Kelch-like protein 12 (KLHL12), which is involved in collagen secretion. LZTR1 could inhibit KLHL12-mediated ubiquitination of SEC31A, a component of coat protein complex II (COPII), whereas LZTR1 deficiency promoted collagen secretion. LZTR1-RIT1 and LZTR1-KLHL12 worked independently regarding molecular interactions and did not directly interfere with each other. Further, we found that LZTR1 deficiency significantly increases lung metastasis and promotes ECM deposition around metastatic tumors. Since collagen-rich extracellular matrix act as pathways for migration and facilitate metastasis, increased expression of RAS and collagen deposition may exert synergistic or additive effects leading to tumor progression and metastasis. In conclusion, LZTR1 deficiency exerts high metastatic potential by enhancing sensitivity to EMT induction and promoting collagen secretion. The functional inhibition of KLHL12 by LZTR1 provides important evidence that LZTR1 may be a repressor of BTB-Kelch family members. These results provide clues to the mechanism of LZTR1-deficiency carcinogenesis.

## Introduction

The RAS subfamily has two forms: the inactive GDP- and active GTP-bound forms. The ratio of active to inactive states is controlled by the guanine nucleotide exchange factor and GTPase-activating protein. Downstream signals, such as mitogen-activated protein kinases (MAPK), are triggered upon activation of the RAS subfamily [1]. However, the effect of quantitative changes in the RAS subfamily on MAPK signaling and its mechanisms are unknown. *Leucine zipper-like transcriptional regulator 1* (*LZTR1*) encodes a BTB-Kelch family member that acts as a substrate adaptor for the CUL3-based ubiquitin E3 ligase complex [2, 3]. LZTR1 targets the RAS subfamily, which includes RIT1, MRAS, HRAS, KRAS, and NRAS [4–7]. LZTR1 regulates polyubiquitination and degradation of the RAS subfamily [6, 7], and LZTR1 dysfunction induces RAS accumulation and activates the MAPK pathway [6, 7]. These findings indicate that the activation levels of the RAS subfamily are partially regulated by ubiquitin-dependent proteasomal degradation.

Mutations in *LZTR1* have been identified in patients with RAS/MAPK signaling pathway-dependent congenital malformation syndrome (RASopathies), glioblastoma, and several cancers via activation of the RAS/MAPK signaling pathway [8–16]. Although recent progress has been made in understanding LZTR1-mediated RAS proteostasis using animal models of RASopathies and leukemogenesis, the influence of LZTR1 deficiency on metastasis is poorly understood. High expression of RIT1, a significant degradation target of the RAS subfamily by the CUL3-LZTR1 complex, induces epithelial-mesenchymal transition (EMT), an early step in tumor metastasis in lung epithelial cells [17]. Conversely, LZTR1 overexpression evokes metastasis-related processes in normal human melanocytes [18]. These reports indicate that LZTR1 may have different roles in each tissue or cancer type and that unknown molecules are involved in LZTR1-related tumor metastasis. However, targets of LZTR1 other than RAS/MAPK-related molecules are not clearly understood. Identification of novel proteins interacting with LZTR1 will help in understanding the molecular function of LZTR1 in tumor metastasis.

Here, we showed that LZTR1 deficiency induces EMT and promotes tumor growth and metastasis in xenograft tumor models. Furthermore, we identified Kelch-like protein 12 (KLHL12) as a novel LZTR1-interacting protein. LZTR1 regulates collagen secretion by suppressing ubiquitination of SEC31A via interaction with KLHL12. Disruption of the interaction between them induces collagen secretion, which is the first step in extracellular matrix (ECM) remodeling, leading to tumor metastasis. These findings provide new insights into the treatment of LZTR1-associated cancers and genetic disorders.

## Materials and methods

### Cell culture

The A549 (CRM-CCL-185) and HEK293 (CRL-1573) cell lines were purchased from ATCC (Manassas, VA, USA). A549 cells were cultured in RPMI 1640 (Thermo Fisher Scientific, Waltham, MA, USA, 21870076) supplemented with 10% fetal bovine serum (FBS), GlutaMAX Supplement (Thermo Fisher Scientific, 35050061), Sodium Pyruvate (Thermo Fisher Scientific, 11360070), MEM Non-Essential Amino Acids Solution (Thermo Fisher Scientific, 11140050), and Antibiotic-Antimycotic (Thermo Fisher Scientific, 15240062) at 37 °C in a 5% CO_2_ atmosphere. HEK293 cells and mouse embryonic fibroblasts (MEFs) were cultured in Dulbecco’s modified Eagle’s medium (DMEM; Thermo Fisher Scientific, 11995073) supplemented with 10% FBS, MEM Non-Essential Amino Acids Solution, and Antibiotic-Antimycotic at 37 °C in a 5% CO_2_ atmosphere.

### Generation of the LZTR1 knockout cell line

A549 cells were transfected with a guide RNA, and the picked clones verified the target sequence, as described previously [7, 19]. The LZTR1 knockout cell line was named A549-KO.

### Small interfering RNA transfection

Cells were transfected with 10 nM siRNA using Lipofectamine RNAiMAX (Thermo Fisher Scientific, 13778150). Here, we used four types of siRNA (GE Healthcare Dharmacon, Lafayette, CO, USA): ON-TARGETplus Non-Targeting Pool (Control-siRNA, D-001810-10-20), ON-TARGETplus SMART Pool-human RIT1 (RIT1-siRNA, L-008390-00-0005), ON-TARGETplus SMART Pool-human Ras Responsive Element Binding Protein 1 (RREB1) (RREB1-siRNA, L-019150-00-0005), and ON-TARGETplus SMART Pool-human LZTR1 (LZTR1-siRNA, L-012318-00-0005).

### Cell invasion assay

A549 cell lines were reverse transfected with Control-siRNA or RIT1-siRNA and seeded at 2 × 10^5^ cells/well in a Corning BioCoat Matrigel Invasion Chamber (Corning, Lowell, MA, USA, 354480), and % invasion was calculated following the manufacturer’s protocol.

### Generation of the *Lztr1*^+/−^ mouse and MEFs

*Lztr1*^+/−^ mice were generated using the CRISPR/Cas9 system, as described previously [20]. The following sequence was selected as gRNA for generation of *Lztr1* deletion (5′-TTCGTGTTCTCAGGACAGAGTGG-3′, where protospacer adjacent motif is underlined). sgRNA was synthesized using CUGA7 gRNA synthesis kit according to the manufacturer’s instructions. Male F0 founder mice bearing mosaic mutations at the target site were crossed with female C57BL/6J mice to produce heterozygous F1 mice. One of the F1 mice harboring a frameshift mutation was backcrossed for more than 10 generations with C57BL/6J mice. Genomic DNA was extracted from the mouse tails using 50 mM NaOH for genotyping. PCR was performed with the following primers: 5′-ATAAGATGTTCGTGTTCTCAGGACAAG-3′ and 5′-CTCCCAAGCCTTATTCCCTTTCCT-3′. The mice were maintained under 12 h light/dark cycles. All animal experiments were performed in accordance with the guidelines for animal experiments at Tohoku University.

E13.5 embryos derived from *Lztr1*^+/−^ × *Lztr1*^+/−^ crosses were dissected by removing the head and internal organs. The collected tissues were homogenized using a 22 G needle for trypsin digestion. Trypsinized cells were washed thrice with PBS and suspended in DMEM containing 10% FBS. The cell suspension was plated in tissue culture flasks to obtain MEFs. All animal experiments were approved by the Animal Care and Use Committee of Tohoku.

### Evaluation of tumor growth and metastasis in vivo

A549 cells were subcutaneously injected into randomized female BALB/c nude mice (Charles River Laboratories Japan, Yokohama, Japan; 1 × 10^6^ cells/mouse). Before the initiation of animal studies, female mice were weighed and randomized for the experiment. The tumor volume was measured using a digital caliper and calculated using the formula: tumor volume = (length × width^2^)/2. For metastasis analysis, we injected A549-WT or A549-KO cells into the tail vein of female BALB/c nude mice using 30 G needles (2 × 10^6^ cells/mouse). After 8 or 12 weeks, the lungs were collected and analyzed. The researchers were not blinded during experiments. The statistical method was not used to predetermine the sample size for the mouse experiments, which was based on previous reports. Conclusions were made based on quantitative parameters and the statistical significance of the data.

### Plasmids

KLHL12, SEC31A, CUL3, ubiquitin (Ub), and KRAS cDNAs were amplified from human cDNA or previously constructed plasmids [7, 13] by PCR with the primer sets shown in Supplementary Table S1 and subcloned into the pENTR-SD-D-TOPO Gateway entry vector (Thermo Fisher Scientific, K242020). These cDNAs were inserted into pcDNA3.1, pcDNA3.2 (V5-tag), or pcDNA-DEST47 (GFP-tag) using Gateway technology. We added each protein tag, and specific mutations were inserted into the plasmid using specific primers and the KOD-Plus-Mutagenesis kit (TOYOBO, Osaka, Japan, SMK-101) (Supplementary Table S1).

### Western blot analyses

Whole-cell lysates were prepared using CelLytic M Cell Lysis Reagent (Sigma Aldrich, St Louis, MO, USA, C2978) or RIPA buffer (50 mM Tris-HCl pH 7.5, 150 mM NaCl, 1% Triton-X, 0.5% sodium deoxycholate, and 0.1% SDS). The transferred membranes were incubated overnight at 4 °C with the following antibodies (catalog numbers in parentheses): HA-tag (3724), E-Cadherin (3195), N-Cadherin (13116), SNAIL (3879), Claudin-1 (13255), GFP (2956), COL1A1 (72026), and GAPDH (2118) purchased from Cell Signaling (Danvers, MA, USA); FLAG-tag (F1804), Monoclonal Anti-Flag M2 peroxidase (HRP) antibody (A8592), RIT1 (HPA053249), and β-actin (A5316) from Sigma Aldrich; LZTR1 (sc-390166) and NRAS (sc-31) from Santa Cruz Biotechnology (Santa Cruz, CA, USA); MRAS (ab176570) and RAS (ab52939) from Abcam (Cambridge, UK); and V5-tag (M215-3), HRAS (18295-1-AP), and KRAS (OP24) from MBL International (MA, USA), Proteintech (Chicago, IL, USA), and Merck Millipore (Billerica, MA, USA), respectively. Original western blots showed in this study are provided in Supplementary Fig. S5.

### Immunofluorescence analyses

A549 cells grown on 13 mm^2^ glass coverslips (Matsunami Glass, Osaka, Japan, C1110) were treated with 10 ng/ml TGF-β1. The fixed cells were stained with anti-N-Cadherin antibody (Cell Signaling Technology, 13116) and ActinGreen 488 (Thermo Fisher Scientific, R37110) [7]. Ascorbate chase analyses were performed by adding ascorbate (0.25 mM ascorbic acid and 1 mM ascorbic acid 2-phosphate) to MEFs from *Lztr1*^+/+^ or *Lztr1*^−/−^ mice, followed by incubation for 0, 10, 30, and 60 min [21]. Cells treated with ascorbate were fixed and stained with anti-COL1A1 antibody (Cell Signaling Technology, 72026).

### Multi-omics analyses

Total RNA and proteins from A549 cells were extracted using TRIzol reagent (Thermo Fisher Scientific, 15596026) following the manufacturer’s protocol. DIA proteomic analyses were performed [22, 23]. Total RNA was analyzed by 3′ RNA-seq using the QuantSeq 3′ mRNA-Seq Library Prep Kit for Illumina (FWD) (Lexogen, Vienna, Austria, 015.384). Multi-omics analysis was performed at the Kazusa DNA Research Institute (Kisarazu, Japan). RNA-seq was quantified with Salmon to calculate transcript abundance [24, 25]. Differential gene expression analysis (DEA) and pathway analysis were performed using edgeR and Metascape.

### Quantitative reverse transcription-PCR (RT-qPCR)

Total RNA preparation and cDNA syntheses were performed using TRIzol reagent and a High-Capacity cDNA Reverse Transcription Kit (Thermo Fisher Scientific, 4374967). RT-qPCR was performed using GoTaq qPCR Master Mix (Promega, WI, USA, A6002) with specific primers (Supplementary Table S2). The mRNA levels were normalized to 18SrRNA levels.

### Immunoprecipitation assay and liquid chromatography-tandem mass spectrometry (LC-MS/MS) analysis

An in vivo ubiquitination assay was performed (7), and HEK293 cells were then transfected with the indicated plasmids. The cell lysates were incubated 48 h later with anti-FLAG M2 Magnetic Beads (Sigma Aldrich, M8823), anti-HA-tag mAb-Magnetic Agarose (MBL International, M132-10), anti-GFP mAb-Magnetic Beads (MBL International, D153-11). The beads were washed four times with wash buffer (50 mM HEPES pH 7.5, 0.15 M NaCl, 0.1% NP-40) and then incubated with SDS sample buffer or 3 × FLAG Peptide (Sigma Aldrich, F4799). To obtain novel proteins interacted with LZTR1, whole-cell lysate from HEK293 cells was immunoprecipitated with an anti-LZTR1 antibody (Santa Cruz Biotechnology, sc-390166) or a control IgG at 4 °C overnight. The immunoprecipitants were analyzed using nano-LC-MS/MS [26].

### Histology and immunohistochemistry (IHC)

Histological and immunohistochemical sections were prepared [27] and stained with hematoxylin and eosin (HE) and Masson’s trichrome (MT) according to standard protocols. For IHC, antigens were activated using a Histofine simple stain kit (Nichirei Biosciences, Tokyo, Japan), and the sections were stained with anti-COL1A1 antibody (Cell Signaling Technology, 72026), anti-*α*-SMA antibody (Cell Signaling Technology, 19245), anti-ZEB1 antibody (Cell Signaling Technology, 70512), anti-SNAIL + SLUG antibody (Abcam, ab180714), and anti-Lamin B1 antibody (Synaptic Systems GmbH, Göttingen, Germany, HS-404013). TUNEL staining was performed using the In Situ Cell Death Detection Kit (Sigma Aldrich, 1684795).

### Statistical analyses

Statistical analyses were performed using R software packages. In vitro and in vivo data are presented as mean ± SD and mean ± SE, respectively. Significant differences were assessed using the Wilcoxon-Mann-Whitney test or Tukey-Kramer test.

## Results

### LZTR1 deficiency-induced high expression of the RAS subfamily and cell growth

**Fig. 1:**
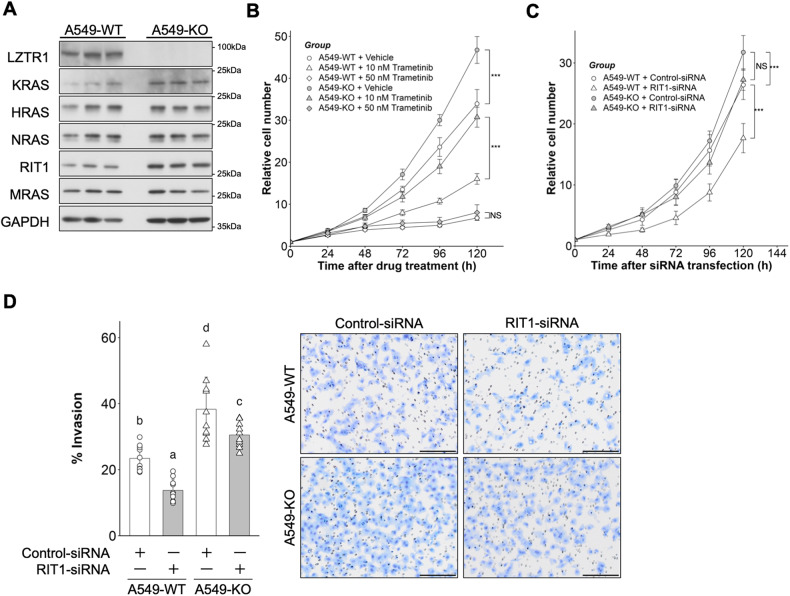
Influence of LZTR1 knockout in A549 cells. **A** A549 cell lines were cultured in 10% serum-containing medium for 48 h, and the expression levels of the RAS family were evaluated by western blot (*n* = 3). **B** Cells were treated with 0.05% DMSO, 10 nM trametinib, or 50 nM trametinib. Cell numbers were quantified at the indicated time points after treatment using Cell Counting Kit-F. Values are the mean ± SD (*n* = 8); *P ≤ 0.05; **P ≤ 0.01; ***P ≤ 0.001 (Wilcoxon-Mann-Whitney test). **C** Cells were transfected with 10 nM Control-siRNA or RIT1-siRNA and cultured in a fresh RPMI medium containing 10% FBS 24 h after transfection. Values are the mean ± SD (*n* = 12); NS not significant; **P ≤ 0.01; ***P ≤ 0.001 (Wilcoxon-Mann-Whitney test). **D** Cells were subjected to the Matrigel invasion assay. Values are the mean ± SD (*n* = 10); Scale bar, 200 µm. Columns not sharing a common letter (a, b, c, d) differ significantly from each other (*P* ≤ 0.05; Tukey-Kramer test).

To investigate the role of LZTR1 in cancer, we generated an LZTR1 knockout A549 cell line, a human lung adenocarcinoma cell line (A549-KO, Supplementary Fig. S1). A549-KO cells expressed high levels of RAS subfamily members, including KRAS and RIT1 (Fig. 1A). We then examined whether LZTR1 degrades KRAS mutants, since A549 cells have a homozygous c.34G>A (p.G12S) mutation. Overexpression of LZTR1 downregulated the expression of several KRAS mutants, including p.G12S (Supplementary Fig. S2). Furthermore, LZTR1 deficiency promoted cell proliferation and invasion (Fig. 1B, C). To examine whether activation of MAPK signaling regulates LZTR1 deficiency-dependent cell growth, we treated the cells with trametinib, a MEK inhibitor. The growth of both cell lines was completely suppressed by treatment with 50 nM trametinib, whereas A549-KO cells were more resistant than A549-WT cells to 10 nM trametinib (Fig. 1B). RIT1 is the most upregulated RAS subfamily member in LZTR1 deficiency in several cancer types [15, 28]. Therefore, we investigated the influence of RIT1 knockdown on LZTR1 deficiency-dependent cellular phenotypes. RIT1 knockdown inhibited cell proliferation in A549-WT cells but not in A549-KO cells. (Fig. 1C). In contrast, RIT1 knockdown suppressed cell invasion in both cell lines, although it did not completely suppress the increase in cell invasion of A549-KO cells (Fig. 1D). These results suggest that LZTR1 regulates the growth and invasion of lung cancer cells through RAS/MAPK signaling. RIT1 accumulation in LZTR1 deficient cells could affect cell invasion, but cell growth might be controlled by the accumulation of other RAS subfamily members, including KRAS p.G12S. From a therapeutic perspective, the reduction of RIT1 may provide benefits in lung cancer therapy with or without *LZTR1* mutations.

### LZTR1 deficiency promoted xenograft tumor growth

**Fig. 2:**
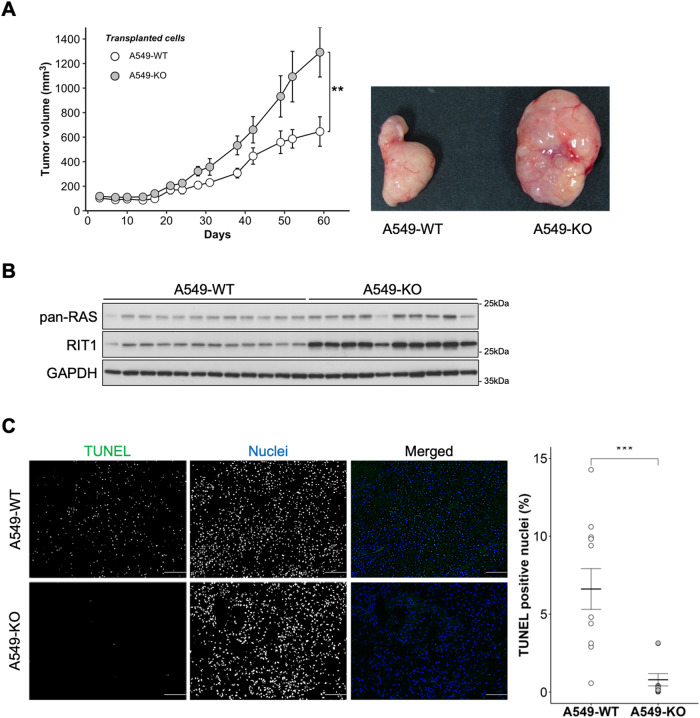
Loss of LZTR1 promotes tumor growth in a mouse xenograft tumor model. **A** A549 cell lines were subcutaneously injected into female BALB/c-nude mice, and the tumors were analyzed. Xenograft tumor volumes were measured at indicated time points. Values are the mean ± SEM; **P ≤ 0.01 (Wilcoxon-Mann-Whitney test, *n* = 12 or 10). **B** Isolated xenograft tumors were lysed using RIPA buffer and their interactions evaluated by western blot analyses. **C** The rate of apoptosis was analyzed by the TUNEL staining; green denotes TUNEL-positive cells; blue denotes nuclei. Values are the mean ± SEM; ***P ≤ 0.001 (Wilcoxon-Mann-Whitney test).

Based on our results and those of previous studies [11, 12, 14] indicating that LZTR1 acts as a tumor suppressor in vitro, we examined whether it also serves as a tumor suppressor in a xenograft tumor model. Therefore, we injected A549-WT or A549-KO cells subcutaneously into BALB/c-nude mice and analyzed the growth rates. The tumor volume of xenografts implanted with A549-KO cells was markedly higher than that of xenografts implanted with A549-WT cells (Fig. 2A). Tumors derived from A549-KO cells showed high expression of RIT1 and low levels of TUNEL-positive nuclei (Fig. 2B, C). We did not detect KRAS expression by western blotting in this model. These data suggest that LZTR1 plays a critical role as a tumor suppressor in vivo.

### Multi-omics analysis demonstrated that LZTR1 deficiency could regulate EMT induction and ECM remodeling

**Fig. 3:**
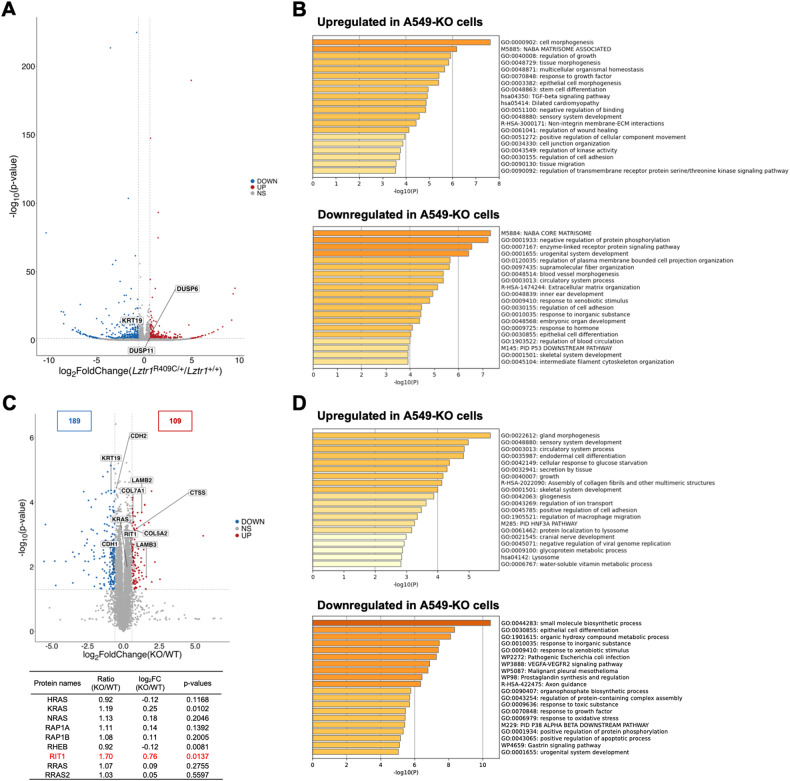
Multi-omics analysis to investigate the LZTR1 knockout influence on lung cancer cell lines. **A** Volcano plot of the data from 3′ RNA-seq is shown (*n* = 3). Differentially expressed genes (DEGs) were identified using a criterion of *P*-values < 0.05 and log_2_ fold change > |0.6|. Genes upregulated or downregulated are shown in red and blue, respectively. **B** Enrichment analyses of multiple DEGs were performed using Metascape, and the relevant enrichment patterns across multiple gene lists and top enriched clusters were represented. **C** Volcano plot of the data from LC-MS/MS proteome analysis (*n* = 3). Differentially expressed proteins (DEPs) were identified using a criterion of *P*-values < 0.05 and log_2_ fold change >|0.6|. Proteins that were upregulated or downregulated are shown in red and blue, respectively. **D** Enrichment analyses of multiple DEPs were performed as in (**B**).

To comprehensively identify the cellular signaling and physiological functions associated with LZTR1 deficiency in cancers, we performed multi-omics analyses using A549-WT and A549-KO cells. A 3′ RNA-seq analysis revealed that LZTR1 deficiency enriched for genes with annotations “cell morphogenesis (GO:0000902)”, “regulation of growth (GO:0040008)”, and cell adhesion-related GO terms in the upregulated gene group. Moreover, *DUSP6* and *DUSP11*, target genes of ERK, were higher in A549-KO cells than in parental cells. These GO terms comport with the known functions of the RAS subfamily or the MAPK signal transduction pathway. Interestingly, “TGF-beta signaling pathway (hsa04350)” was also enriched in the upregulated gene group (Fig. 3B). This pathway induces EMT, which is a critical process in tumor progression and metastasis. Conversely, the downregulated gene group in A549-KO cells demonstrated enriched GO terms, including “negative regulation of protein phosphorylation (GO:0001933)”, “enzyme-linked receptor protein signaling pathway (GO:0007167)”, and “epithelial cell differentiation (GO:0030855)” (Fig. 3B).

Proteomic analysis revealed that LZTR1 deficiency markedly downregulated 189 and upregulated 109 proteins with RIT1 the most upregulated RAS subfamily member (about 1.7-fold increase) (Fig. 3C). KRAS protein levels increased by 1.2-fold, whereas other RAS members remained unchanged in lung adenocarcinoma cells. Functional enrichment analysis of differentially expressed proteins (DEPs) revealed that “epithelial cell differentiation (GO:0030855)” was enriched in the downregulated DEP group, similar to the transcriptomic analysis (Fig. 3D). The GO terms contained EMT-related molecules, and an increase in RIT1 can promote expression changes in EMT markers, including downregulation of keratin-19 (KRT19) [17]. Consistent with the report, KRT19 expression was reduced by about half in A549-KO cells (Fig. 3C). Furthermore, “Assembly of collagen fibrils and other multimeric structures (R-HSA-2022090)” term was enriched in the upregulated DEP group. This term contains proteins, including Cathepsin S (CTSS), LAMB2, LAMB3, COL5A2, and COL7A1, which are lysosomal protease or ECM components (Fig. 3C). Secreted Cathepsin modifies the tumor microenvironment through ECM remodeling, which occurs in the early phase of metastasis [29, 30]. These data suggest that LZTR1 deficiency regulates EMT induction and ECM remodeling, leading to tumor metastasis.

### LZTR1 deficiency promoted the induction of EMT

**Fig. 4:**
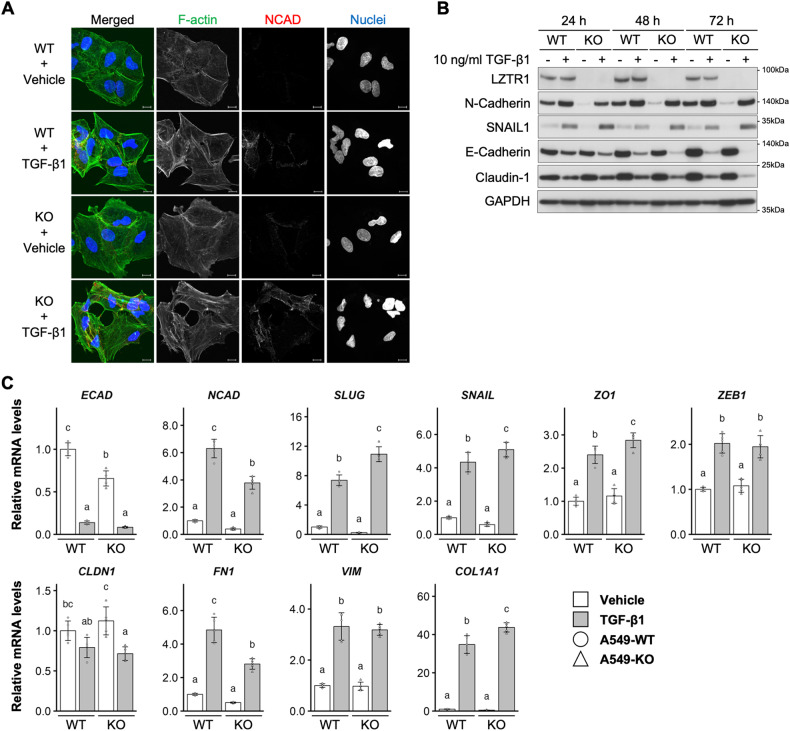
LZTR1 knockout influence on EMT induction. **A** Cells were treated with TGF-β1 for 72 h and stained with anti-N-cadherin (NCAD) antibody (red), ActinGreen 488 (F-actin, green), and NucBlue Stain (nuclei, blue). Scale bar, 10 µm. **B** Cells were treated with TGF-β1 for the indicated times and their interactions evaluated by western blot analyses. **C** Cells were treated with 10 ng/ml TGF-β1 for 72 h. Total RNA was collected and subjected to RT-qPCR (*n* = 5).

Next, we investigated the effect of LZTR1 deficiency on EMT induction in lung cancer cells. Treating A549 cells with TGF-β1 induces EMT, wherein cells lose epithelial morphology and gain mesenchymal hallmarks, including high expression of N-Cadherin and stress fibers [31]. Indeed, TGF-β1 treatment induced EMT-like morphological changes in A549-WT and A549-KO cells. However, there was no apparent difference in these changes 72 h after TGF-β1 treatment (Fig. 4A). EMT-associated morphological changes were observed in A549-KO cells but not in A549-WT cells 24 h after TGF-β1 treatment (Supplementary Fig. S3A).

We then assessed the effect of LZTR1 knockout on chronological changes in EMT-related marker proteins. Upregulation of N-Cadherin was observed in both cell lines. Basal levels were lower in A549-KO cells than in A549-WT cells (Fig. 4B). The expression of SNAIL, an EMT-related transcription factor, was higher in A549-KO cells than in A549-WT 24-72 h after TGF-β1 treatment (Fig. 4B). Conversely, the loss of LZTR1 markedly decreased the expression of E-Cadherin and Claudin-1 compared to that in control cells (Fig. 4B and Supplementary Fig. S3B). It is well known that these markers and EMT show inverse correlation [32, 33]. Similarly, the mRNA levels of EMT-related marker genes, including *SNAIL1*, *SLUG*, *ZO-1*, and *COL1A1*, were significantly higher in A549-KO cells than in A549-WT cells 72 h after TGF-β1 treatment (Fig. 4C). These results indicate that LZTR1 deficiency promoted TGF-β1-mediated EMT induction.

RAS-responsive element-binding protein 1 (RREB1) is a transcription factor involved in the induction of RAS/MAPK-related EMT [34, 35]. Thus, we investigated whether RREB1 participates in LZTR1 deficiency-mediated EMT induction. As shown in Supplementary Fig. S3C, the mRNA levels of EMT-related marker genes increased in A549-KO cells 24 h after TGF-β1 treatment, similar to that at 72 h (Fig. 3C). LZTR1 deficiency-dependent increases in *SLUG* and *ZEB1* were completely eliminated by RREB1 knockdown (Supplementary Fig. S3C). However, the increase in expression of other EMT marker genes was not suppressed by RREB1 knockdown (Supplementary Fig. S3C). These results suggest that LZTR1 deficiency can promote EMT induction and that there are other pathways besides RREB1.

### LZTR1-regulated collagen secretion by inhibiting the KLHL12-mediated SEC31A ubiquitination

**Fig. 5:**
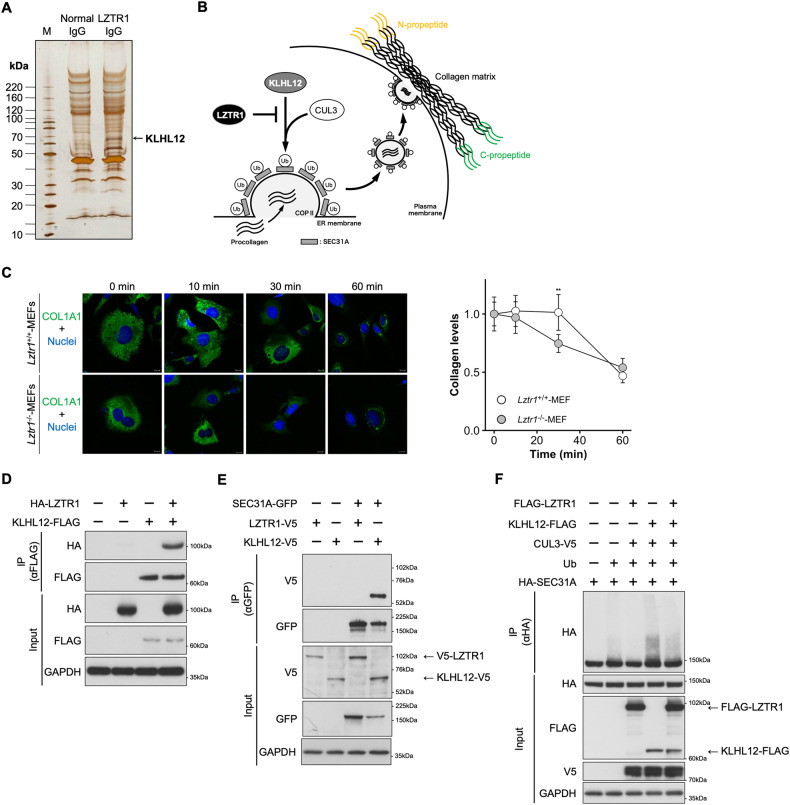
LZTR1 indirectly regulates SEC31A ubiquitination through the inhibition of KLHL12 function. **A** The results of liquid chromatography-tandem mass spectrometry. **B** How LZTR1 regulates SEC31A ubiquitination and inhibits collagen secretion through the inhibition of KLHL12 function. **C** Ascorbate chase analyses were performed by adding ascorbate (0.25 mM ascorbic acid and 1 mM ascorbic acid 2-phosphate) to MEFs from *Lztr1*^+/+^ or *Lztr1*^−/−^ mice. Cells were stained with anti-COL1A1 antibody (green) and NucBlue Stain (nuclei, blue). Scale bar, 20 µm. Values are the mean ± SD; **P ≤ 0.01 (Wilcoxon-Mann-Whitney test, *n* = 5). **D**, **E** HEK293 cells were transfected with the indicated expression plasmids, and the lysates were subjected to co-immunoprecipitation assays using anti-FLAG M2 Magnetic Beads or anti-GFP mAb-Magnetic Beads. The immunoprecipitants were subjected to western blot analyses. **F** An in vivo ubiquitination assay was performed with anti-HA-tag mAb-Magnetic Agarose. The ubiquitination status of SEC31A was evaluated by western blot.

The results of the proteomic analysis suggest that ECM remodeling is affected by LZTR1 deficiency. To clarify the relationship between this deficiency and ECM remodeling, we searched for novel proteins that interact with LZTR1. Immunoprecipitants with anti-LZTR1 antibodies were analyzed by LC-MS/MS and the proteins that interacted with LZTR1 were identified (Fig. 5A). Kelch-like protein 12 (KLHL12), a novel interactor, is a member of the BTB-Kelch family and regulates collagen secretion through ubiquitination of SEC31A, a component of the outer layer of coat protein complex II (COPII). COPII transports collagen from the endoplasmic reticulum to the Golgi apparatus [3, 21, 36]; therefore, we hypothesized that LZTR1 is associated with collagen secretion via its interaction with KLHL12.

Next, we examined the effect of LZTR1 deficiency on collagen secretion in MEFs from *Lztr1* knockout mice. Under the stimulation of collagen secretion with ascorbic acid, the rate of COL1A1 secretion was faster in *Lztr1*^−/−^ MEFs than in *Lztr1*^+/+^ MEFs (Fig. 5C). Co-immunoprecipitation assays showed that LZTR1 interacted with KLHL12 but not with SEC31A in HEK293 cells transfected with indicated plasmids. (Fig. 5D, E). Immunofluorescence analysis showed that LZTR1 intracellular colocalized with KLHL12 (Supplementary Fig. S4A). The pulled-down SEC31A proteins showed two bands, of which the lower band was more intense in co-expressing LZTR1 than KLHL12 (Fig. 5E). Conversely, in SK-N-SH whole-cell lysates, LZTR1 knockdown decreased the lower band of SEC31A and increased the upper band (Supplementary Fig. S4B). From these results, we predicted that the upper and lower bands are ubiquitinated and non-ubiquitinated SEC31A, respectively. We then performed an in vivo ubiquitination assay to determine the influence on ubiquitination levels of SEC31A by LZTR1. SEC31A ubiquitination increased in cells expressing KLHL12, whereas this increase was suppressed by LZTR1 co-expression, as expected (Fig. 5F). In contrast, KLHL12-mediated SEC31A ubiquitination was enhanced by LZTR1 knockdown (Supplementary Fig. S4C). These results suggest that LZTR1 controls collagen secretion, the first step in ECM remodeling, via indirectly regulating SEC31A ubiquitination. Furthermore, LZTR1-dependent modification to KLHL12/SEC31A was independent of the introduction of wild-type RIT1 or an activated mutant (p.M90I) (Supplementary Fig. S4D–F), and KLHL12 did not affect the RIT1 ubiquitination by LZTR1 (Supplementary Fig. S4G). From these results, LZTR1-RIT1 and LZTR1-KLHL12 work independently regarding molecular interactions and do not directly interfere with each other.

### LZTR1 deficiency enhanced metastasis of lung cancer cells in vivo

**Fig. 6:**
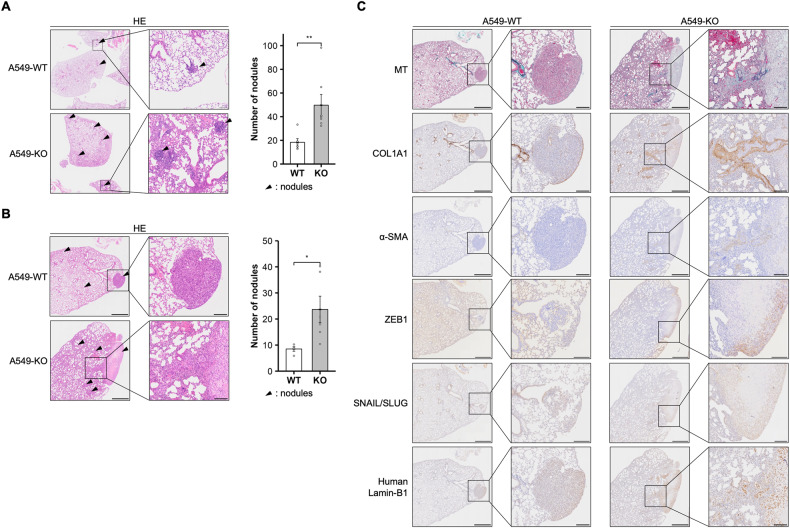
LZTR1 loss promotes tumor metastasis in a metastatic xenograft model. **A**, **B** A549 cell lines were tail vein injected into female BALB/c-nude mice. The level of metastasis to the lungs was assessed at (**A**) 8 or (**B**) 12 weeks after injection (*n* = 4–7). The collected lungs were stained with hematoxylin and eosin stain (HE). Arrowheads indicate representative metastatic nodules. Values are the mean ± SEM; **P ≤ 0.01 (Wilcoxon-Mann-Whitney test). **C** The collected lungs were stained with Masson’s trichrome stain (MT), anti-COL1A1 antibody, anti-*α*-SMA antibody, anti-ZEB1 antibody, anti-SNAIL + SLUG antibody, and anti-human specific Lamin-B1 antibody.

EMT induction and ECM secretion occur in the early phases of tumor metastasis. To investigate the influence of LZTR1 deficiency on tumor metastasis, we injected A549-WT or A549-KO cells into the tail vein of BALB/c nude mice and examined their lung metastasis potential. At 8- and 12-weeks post-cell administration, the mice injected with A549-KO cells showed significant increases in lung metastatic nodules (Fig. 6A, B). Moreover, MT staining and immunohistochemical staining of COL1A1 demonstrated newly synthesized ECM around and inside the A549-KO metastatic tumors (Fig. 6C). IHC showed that alpha-smooth muscle actin (*α*-SMA), a marker of EMT and stromal myofibroblast, ZEB1, and SNAIL/SLUG-positive area were increased around and/or inside the metastatic tumors derived from A549-KO cells (Fig. 6C). These data indicated that LZTR1 deficiency could promote metastasis and ECM deposition.

## Discussion

Here, we demonstrated that the loss of LZTR1 could induce the accumulation of RIT1 and KRAS in lung adenocarcinoma cells and promote tumor growth and metastasis. As a molecular mechanism of tumor metastasis promotion due to LZTR1 deletion, we showed that LZTR1 deficiency promotes EMT induction and ECM deposition in vitro and in vivo. In the ECM deposition analysis, we identified KLHL12 related to collagen secretion as a novel interacting protein with LZTR1. KLHL12-mediated SEC31A ubiquitination was suppressed by LZTR1, whereas LZTR1 deficiency induced collagen secretion. The functional inhibition of KLHL12 by LZTR1 indicated that LZTR1 may act as a suppressor of the BTB-Kelch family. These findings revealed a novel function of LZTR1 in lung cancer progression.

BTB-Kelch family members generally homodimerize and recognize one substrate per molecule directly. Several members, such as KLHL9/KLHL13, are known to form heterodimers [37], but the molecular details of how these heterodimers engage with their substrates have yet to be established. The protein structure of LZTR1 (an N-terminal Kelch motif, followed by two BTB-back domains) differs from that of most other BTB-Kelch members (an N-terminal BTB-back domain, followed by a Kelch motif). LZTR1 interacts with CUL3 through both BTB-back domains [6], whereas other BTB-Kelch family members interact with CUL3 through only one BTB domain. Thus, LZTR1 may homo- or hetero-multimerize complexes with the BTB-Kelch family to form a more complicated CUL3 complex that recognizes a variety of substrates. However, it has not been reported whether LZTR1 interacts with other members of the BTB-Kelch family. Here, we identified several LZTR1-interacting proteins, including KLHL12 (a BTB-Kelch protein) and revealed that LZTR1 deficiency promotes collagen secretion by inhibiting KLHL12-mediated SEC31A ubiquitination (Fig. 5). The fact that LZTR1 did not bind to SEC31A suggests that LZTR1 competes with KLHL12-mediated ubiquitination via heterodimerization with KLHL12. Our results indicate that LZTR1 indirectly prevents the ubiquitination of substrates of other BTB-Kelch members. Mutations in *CUL3* or its substrate adaptor proteins have been identified in patients with congenital heart disease, developmental delay, cranio-lenticulo-sutural dysplasia, and cancers [38–41]. Therefore, the loss of LZTR1 may be involved in the pathogenesis of various diseases through the functional inhibition of other BTB-Kelch family members. Our discovery of an indirect substrate of LZTR1 is important for elucidating the pathogenesis of diseases caused by mutations in *LZTR1*.

The observed increase in tumor metastasis and collagen deposition due to LZTR1 deficiency is valuable for understanding the influence of *LZTR1* mutations on cancers (Fig. 6). Cancer metastasis involves multiple steps, including the closely related EMT induction and ECM remodeling [42]. In the analysis of EMT induction, LZTR1 deficiency significantly increased the expression of various EMT markers including SLUG and SNAIL. These markers are immediate early response factors in TGF-β1-mediated EMT [43]. Indeed, 24 h after TGF-β1 treatment, an increase in the mRNA levels of EMT markers and EMT-associated morphological changes were observed in LZTR1 knockout cells (Supplementary Fig. S3A, C). These results suggest that LZTR1 deficiency enhances sensitivity to EMT induction in the presence of TGF-β1. This is the first evidence of a relationship between LZTR1 and EMT.

ECM deposition analysis showed that LZTR1 deficiency promoted collagen secretion and induced high expression of *COL1A1* in the presence of TGF-β1, while the basal level of *COL1A1* in A549-KO cells was lower than that in the control cells (Fig. 4C). COL1A1, an ECM member, is an independent prognostic factor that promotes EMT in lung cancers via the TGF-β1 signaling pathway [44, 45]. Moreover, procollagen C-propeptides contribute to the negative feedback loop of collagen gene expression and enhance TGF-β signaling [46–48], findings consistent with the changes in COL1A1 expression observed here. Thus, LZTR1 deficiency may regulate EMT induction through collagen secretion. As shown in Supplementary Fig. S4, increased expression of RAS and KLHL12/SEC31A-mediated collagen secretion in LZTR1 deficiency were independent regarding molecular interactions. Since collagen-rich extracellular matrix act as pathways for migration and facilitate metastasis [49, 50], increased expression of RAS and collagen deposition may exert synergistic or additive effects leading to tumor progression and metastasis. However, the detailed relationship between increased expression of RAS and collagen secretion in LZTR1 deficient tumors remains unclear. Future studies are needed to clarify whether these independent targets of LZTR1 exert synergistic or additive effects.

There is growing evidence that the RAS subfamily is a target molecule for CUL3-LZTR1-dependent polyubiquitination; however, the mechanism through which LZTR1 recognizes its target substrates remains unclear. LZTR1 deficiency increased RIT1 and KRAS expression in lung adenocarcinoma cells (Fig. 3C). In contrast, LZTR1 deficiency increased RIT1, KRAS, MRAS, and NRAS expression in fetal liver and acute monocytic leukemia cells [15]. From these results and those of a previous report, it is shown that RIT1 expression is increased by LZTR1 deficiency in various cell lines, while the changes in classical RAS and MRAS expression may differ in each cell and tissue type. These differences indicate that unknown molecules are involved in the RAS subfamily and other substrate recognition by the LZTR1-CUL3 complex. The finding that LZTR1 forms heterodimers with BTB-Kelch proteins, such as KLHL12, may provide clues to understanding substrate recognition by LZTR1; however, the detailed mechanisms by which LZTR1 recognizes its target substrates need investigating.

In summary, the loss of LZTR1 promoted tumor growth and metastasis through the activation of the RAS/MAPK signaling pathway, EMT induction, and ECM deposition. Identifying an indirect target of LZTR1 provides important evidence that LZTR1 could be a functional repressor of BTB-Kelch family members. More insight into its pathophysiological contributions is needed to develop effective interventions for patients harboring *LZTR1* variants. Our findings provide novel insights into the treatment of LZTR1-related tumors and other genetic disorders.

## Supplementary information

Supplemental Material

Original Data File

Reproducibility checklist

## Data Availability

All data analyzed during this study are available from the corresponding author upon reasonable request.
