# Supplementary material for: LZTR1 deficiency exerts high metastatic potential by enhancing sensitivity to EMT induction and controlling KLHL12-mediated collagen secretion
Source: Cell Death Dis. 2023 Aug 25;14(8):556. doi: 10.1038/s41419-023-06072-9 (PMC10457367; doi:10.1038/s41419-023-06072-9)
Supplement: Supplementary file 1 — Supplemental Material [file 41419_2023_6072_MOESM1_ESM.pdf]

Title

LZTR1 deficiency exerts high metastatic potential by enhancing sensitivity to EMT induction and controlling KLHL12-mediated collagen secretion

Author Names: Taiki Abe[1,4*], Shin-ichiro Kanno[2], Tetsuya Niihori[1], Miho Terao[3], Shuji Takada[3], Yoko Aoki[1*]

Affiliations

[1] Department of Medical Genetics, Tohoku University School of Medicine, Sendai, Japan

[2] Division of Dynamic Proteome, Institute of Development, Aging, and Cancer, Tohoku University, Sendai, Japan

[3] Department of Systems BioMedicine, National Research Institute for Child Health and Development, Tokyo, Japan

[4] Lead contact

*Correspondence should be addressed to Taiki Abe, Ph.D. and Yoko Aoki, MD, Ph.D. Department of Medical Genetics, Tohoku University School of Medicine, Sendai 980-8574, Japan

Email: Taiki Abe, abet@med.tohoku.ac.jp; Yoko Aoki, aokiy@med.tohoku.ac.jp, Phone: +81-22-717-8137, Fax: +81-22-717-8142

This PDF file includes: Supplementary Figures, and Supplementary Tables.

Supplementary Figures

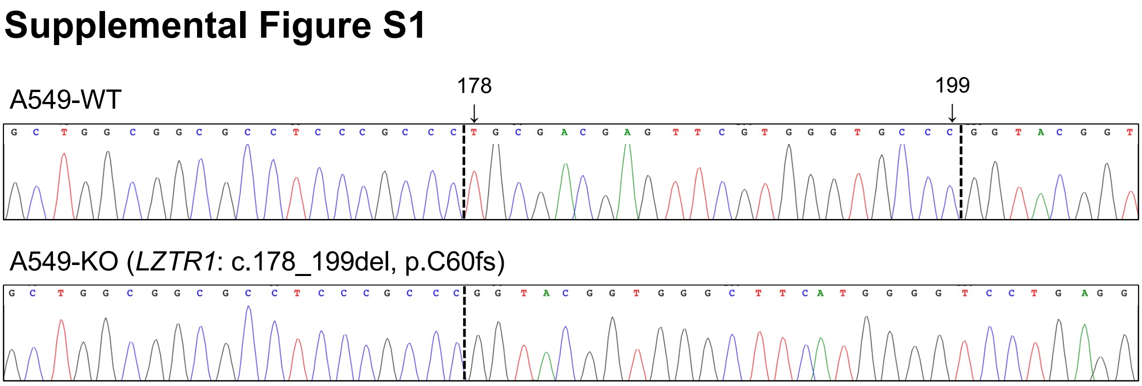

Supplementary Figure S1.

Sequence analysis of parental cells (A549-WT) and A549-KO cells.

Supplemental Figure S2

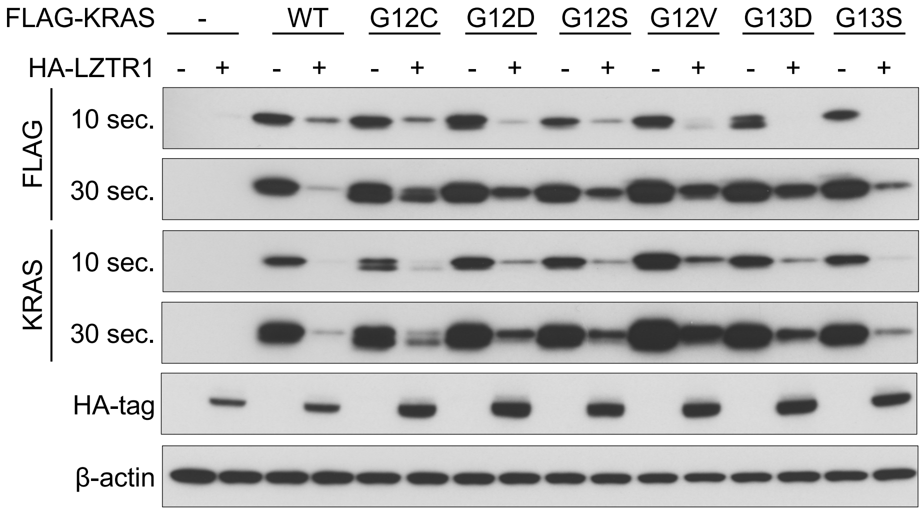

Supplementary Figure S2.

HEK293 cells were transfected with HA-LZTR1 and indicated FLAG-KRAS plasmids and RAS expression levels evaluated.

Supplemental Figure S3

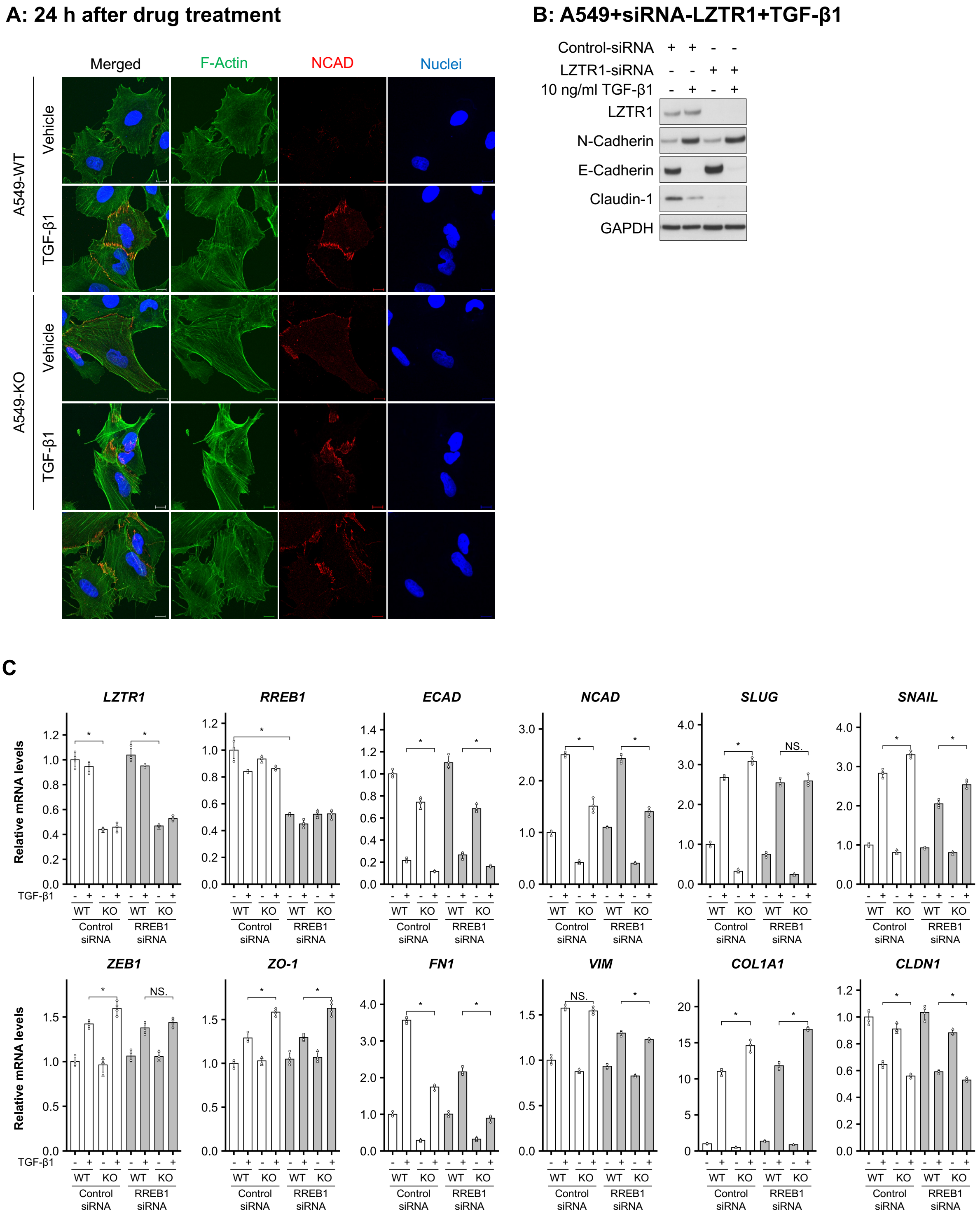

Supplementary Figure S3.

(A) Cells were treated with TGF-β1 for 24 h and stained with anti-N-cadherin (NCAD) antibody (red), ActinGreen 488 (F-actin, green) and NucBlue Stain (nuclei, blue). Scale bar, 10 μm. (B, C) A549 cell lines were transfected with 10 nM Control-siRNA, LZTR1-siRNA, or RREB1-siRNA. Cells were treated 24 h later with 10 ng/ml TGF-β1 for 24 h and the expression levels of EMT marker proteins were evaluated by western blot or RT-qPCR.

Supplemental Figure S4

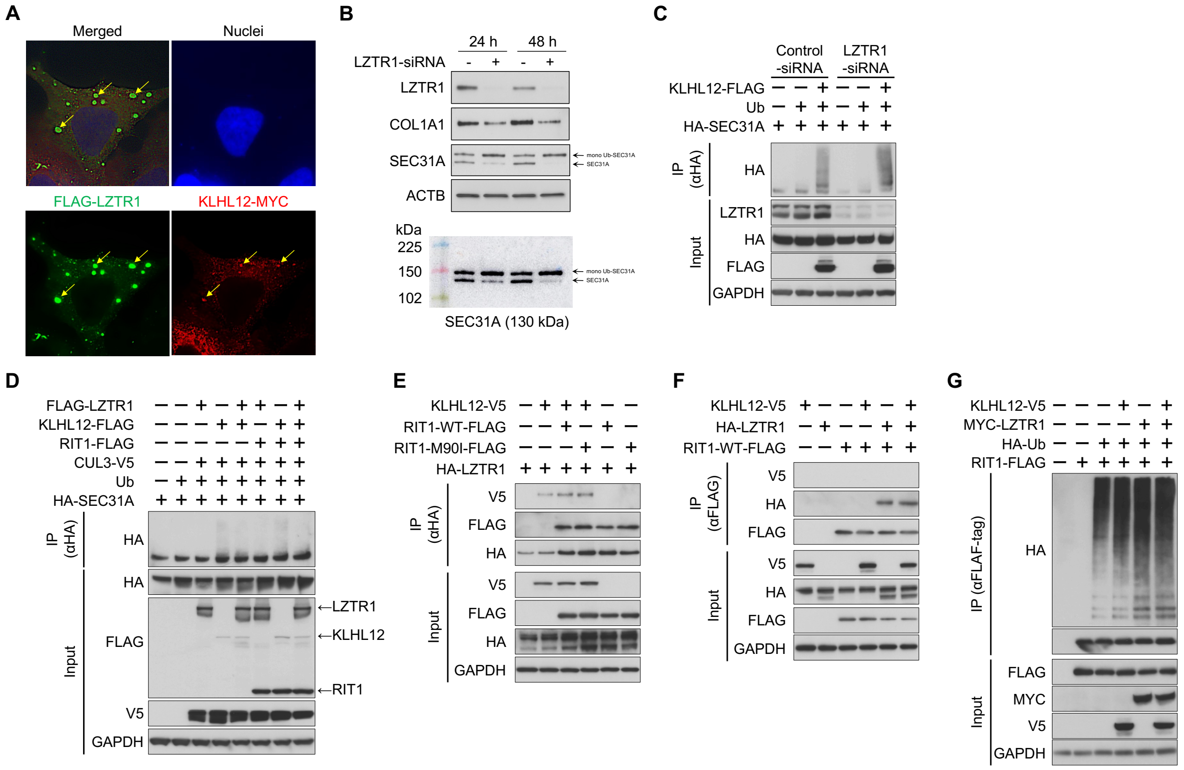

Supplementary Figure S4.

(A) HEK293 cells grown on 13 mm^2 glass coverslips (Matsunami Glass, Osaka, Japan) were transfected with FLAG-LZTR1 and KLHL12-Myc plasmids. The fixed cells were incubated in PBS containing 0.1% Triton X-100 for 10 minutes and incubated in BlockAid Blocking solution (Thermo Fisher Scientific) for 1 h at room temperature. The coverslips were incubated overnight at 4°C with primary antibodies diluted in Can Get Signal Immunostain Immunoreaction Enhancer Solution (Toyobo), and further incubated with secondary antibodies for 1 h at room temperature. The yellow arrows indicate representative locations where LZTR1 and KLHL12 overlap. (B) SK-N-SH cells were transfected with 10 nM Control-siRNA or LZTR1-siRNA. The expression levels of LZTR1, COL1A1, SEC31A, and ACTB were evaluated 24 or 48 h after transfection by western blot. (C) HEK293 cells were transfected with indicated plasmids and siRNAs. Cell extracts were subjected to in vivo

ubiquitination assay. (D) The influence of RIT1 expression on LZTR1-mediated KLHL12/SEC31A modification was evaluated by in vivo ubiquitination assay using denatured cell lysates and anti-HA mAb-Magnetic Beads. (E, and F) HEK293 cells were transfected with the indicated expression plasmids, and the lysates were subjected to co-immunoprecipitation assays using anti-FLAG M2 Magnetic Beads or anti-HA mAb-Magnetic Beads. The immunoprecipitants were subjected to western blot analyses. (G) The influence of KLHL12 on LZTR1-mediated RIT1 ubiquitination was evaluated by in vivo ubiquitination assay using denatured cell lysates and anti-FLAG M2 Magnetic Beads.

Supplementary Table 1. Primers used for construct preparation.

Construct name/mutated position	Forward primer (5´ to 3´)	Reverse primer (5´ to 3´)
HA-LZTR1	TGGAACATCGTATGGGTACATGGTGAAGGGCTCCTTCTTAAAG	GATTACGCTGGTGGAGGTGGATCAGCTGGACCGGGC
KLHL12	CACCATGGGAGGCATTATGGCCCC	CTTCTCGCGGAGAACAC
KLHL12-FLAG	gacatcgactacaaggatgacgatgacaagTAGTGAAAGGGTGGGCGCGCCGACCCAGCTTTCTTG	atgatctttataatcaccgtcatggtctttgtagtcCTTCTCGCGGAGAACACAAACACCAGCATC
SEC31A	CACCATGAAGTTAAAGGAAGTAGATCGTACAGCC	GACACCCAGCTTATTGGCCTGGG
FLAG-KRAS	GGTGGAGGTGGATCAATGACTGAATATAAACTTGTGGTAGTTGG	CTTGTCATCGTCGTCCTTGTAGTCCATGGTGAAGGGCTCCTTCTTAAAG
FLAG-KRAS-G12C	TGTGGCGTAGGCAAGAGTGCC	AGCTCCAACTACCACAAGTTTATATTCAG
FLAG-KRAS-G12D	ATGGCGTAGGCAAGAGTGCCTTG	CAGCTCCAACTACCACAAGTTTATATTC
FLAG-KRAS-G12S	AGTGGCGTAGGCAAGAGTGCCTTG	AGCTCCAACTACCACAAGTTTATATTCAG
FLAG-KRAS-G12V	TTGGCGTAGGCAAGAGTGCCTTGACG	CAGCTCCAACTACCACAAGTTTATATTCAG

FLAG-KRAS-G13D	ACGTAGGCAAGAGTGCCTTGACGATAC	CACCAGCTCCAACTACCACAAG
FLAG-KRAS-G13S	AGCGTAGGCAAGAGTGCCTTGACG	ACCAGCTCCAACTACCACAAG

Supplementary Table 2. Primers for RT-qPCR.

Genes	Forward primer (5´ to 3´)	Reverse primer (5´ to 3´)
18S rRNA	ACCGCGGTTCTATTTTGTTG	AGTCGGCATCGTTTATGGTC
LZTR1	GGTGGAGACAATGGGAAGACC	GTGGGGTCCCAGTGGTAAAG
RREB1	CCCGGCCGTTGCTCC	TCTCTTGGTAGTACTCGTTGACA
CDH1 (*ECAD*)	TGAGCACGTGAAGAACAGCA	GGTATGGGGGCGTTGTCATT
CDH2 (*NCAD*)	TCGAAGGATGTGCATGAAGGA	GCAGGCTCACTGCTCTCATA
SNAIL	CTAGGCCCTGGCTGCTACAA	GACATCTGAGTGGGTCTGGAG
SLUG	CATGCCTGTCATACCACAAC	GGTGTCAGATGGAGGAGGG
ZEB1	GACAGTGTTACCAGGGAGGAG	ACTCGCATTCATCATCTTTTACTGT
ZO-1	GTCCAGAATCTCGGAAAAGTGCC	CTTTCAGCGCACCATACCAACC
VIM	GCCGAAAACACCCTGCAATC	GCGTTCAAGGTCAAGACGTG
CLDN1	GGGCAGATCCAGTGCAAAGT	GAGGATGCCAACCACCATCA
FN1	CCCTGGTGTCACAGAGGCTA	GTTGGGGAAGCTCGTCTGTC
COL1A1	GGCTCCTGCTCCTCTTAGC	GGTTTCCACACGTCTCGGTC
