# Supplementary material for: LZTR1 deficiency exerts high metastatic potential by enhancing sensitivity to EMT induction and controlling KLHL12-mediated collagen secretion
Source: Cell Death Dis. 2023 Aug 25;14(8):556. doi: 10.1038/s41419-023-06072-9 (PMC10457367; doi:10.1038/s41419-023-06072-9)

Supplementary Figure S5: Original western blots

Original western blots: Figure 1A

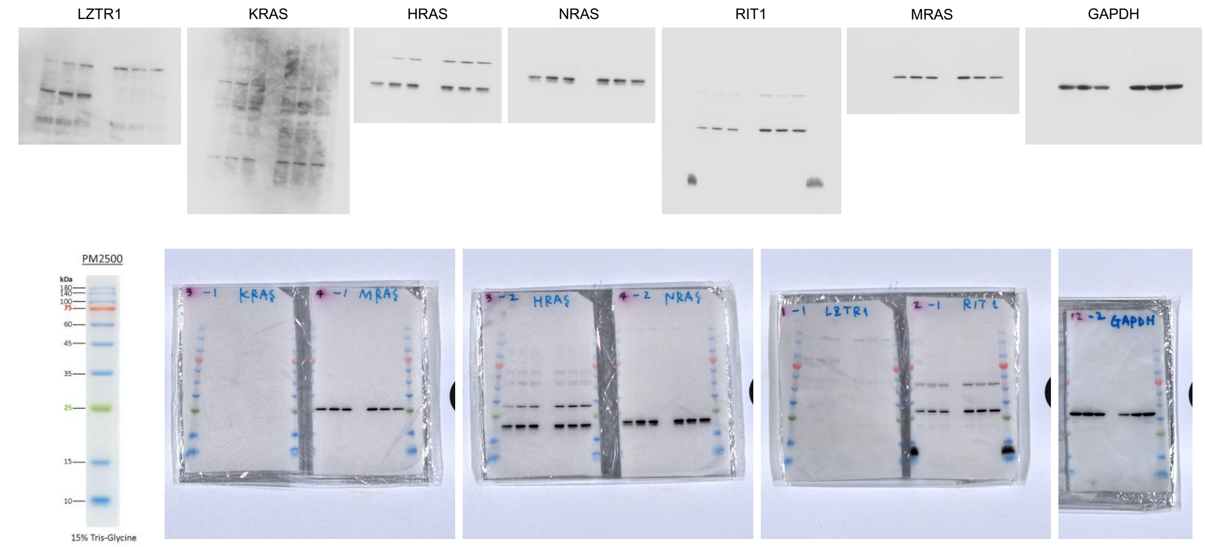

ExcelBand 3-color Regular Range Protein Marker (SMOBIO #PM2500)
URL:https://www.cosmobio.co.jp/product/detail/excelband-protein-ladder-smo.asp?entry_id=13990

Original western blots: Figure 2B

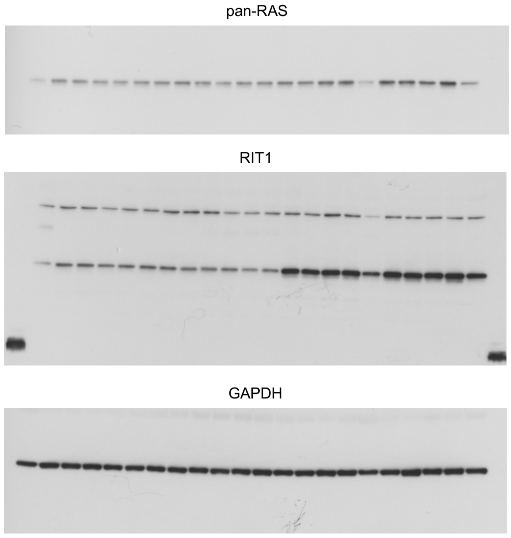

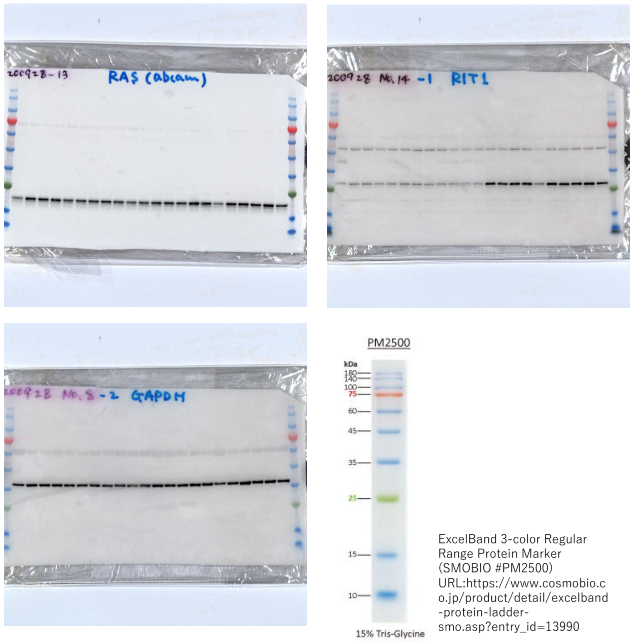

ExcelBand 3-color Regular Range Protein Marker (SMOBIO #PM2500) URL:https://www.cosmobio.co.jp/product/detail/excelband-protein-ladder-smo.asp?entry_id=13990

Original western blots: Figure 4B

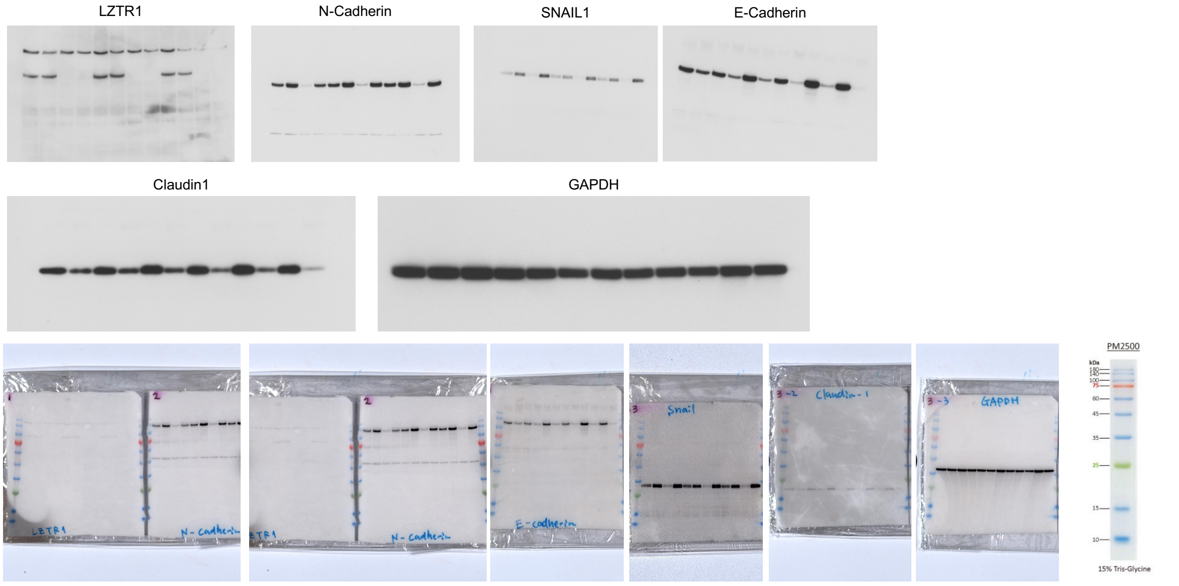

ExcelBand 3-color Regular Range Protein Marker (SMOBIO #PM2500)
URL:https://www.cosmobio.co.jp/product/detail/excelband-protein-ladder-smo.asp?entry_id=13990

Original western blots: Figure 5

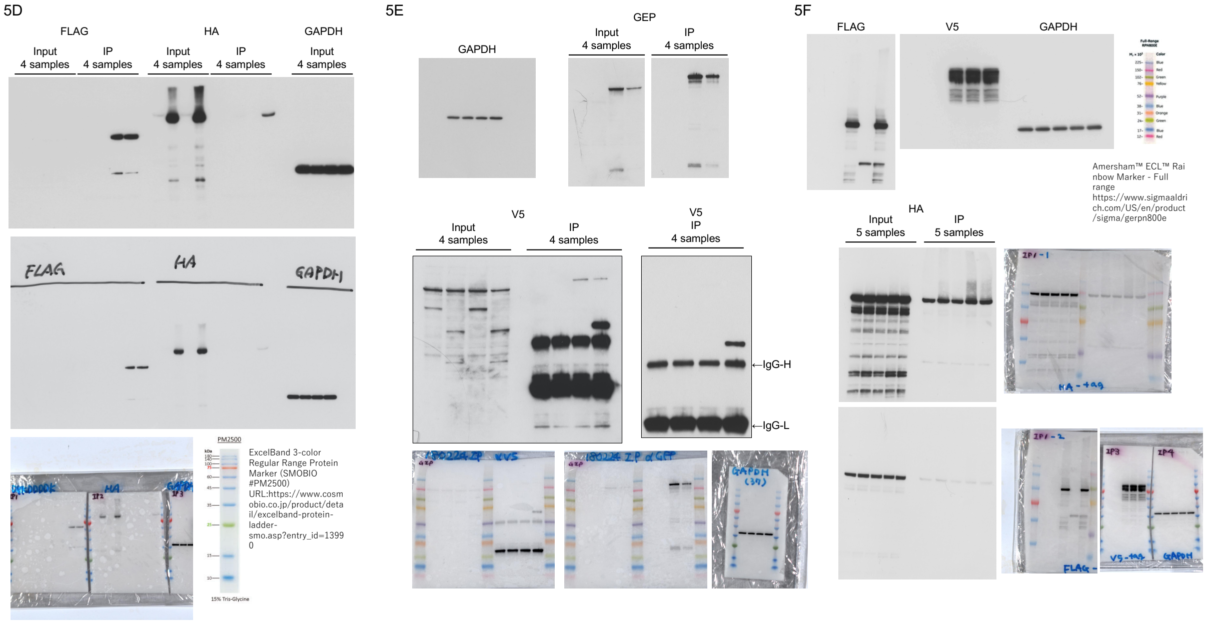

Supplement: Supplementary file 2 — Original Data File [file 41419_2023_6072_MOESM2_ESM.pdf]
